# EphA4 is a prognostic factor in gastric cancer

**DOI:** 10.1186/1472-6890-13-19

**Published:** 2013-06-05

**Authors:** Kohji Miyazaki, Mikito Inokuchi, Yoko Takagi, Keiji Kato, Kazuyuki Kojima, Kenichi Sugihara

**Affiliations:** 1 Department of Surgical Oncology, Tokyo Medical and Dental University, 1-5-45, Yushima, Bunkyo-ku, Tokyo 113-8519, Japan; 2 Department of Translational Oncology, Tokyo Medical and Dental University, 1-5-45, Yushima, Bunkyo-ku, Tokyo, 113-8519, Japan; 3 Department of Minimally Invasive Surgery, Tokyo Medical and Dental University, 1-5-45, Yushima, Bunkyo-ku, Tokyo, 113-8519, Japan

**Keywords:** EphA4, EphA2, ephrinA1, Gastric cancer

## Abstract

**Background:**

Erythropoietin-producing hepatocellular (Eph) receptor, consisting of a family of receptor tyrosine kinases, plays critical roles in tumour development and is considered an attractive target for cancer therapy.

**Methods:**

Tumour samples were obtained from 222 patients with gastric adenocarcinoma who underwent gastrectomy. The expressions of EphA2, EphA4, and ephrinA1 were evaluated immunohistochemically.

**Results:**

High expressions of EphA2, EphA4, and ephrinA1 significantly correlated with variables related to tumour progression, including the depth of invasion, metastatic lymph nodes, pathological stage, and distant metastasis or recurrent disease. High expressions of EphA2, EphA4, and ephrinA1 were significantly associated with poorer disease-specific survival (DSS; p < 0.001, p < 0.001, p = 0.026). On multivariate analysis, EphA4 was an independent prognostic factor of DSS (hazard ratio [HR], 2.3; 95% confidence interval [CI], 1.1-4.8; p = 0.028), and EphA2 tended to be a prognostic factor (HR, 2.4; 95% CI, 1.0-5.8; p = 0.050). In stage II and III cancer, EphA4 and EphA2 were both significantly associated with shorter survival (p = 0.007 and 0.019), but only EphA2 was an independent prognostic factor (HR, 2.6; 95% CI, 1.1-6.3; p = 0.039).

**Conclusion:**

EphA4 may play important roles in tumor progression and outcomes in patients with gastric cancer.

## Background

Gastric cancer is the fourth most common malignancy and the second leading cause of death in the world [1]. The outcomes of gastric cancer remain poor, with an estimated relative 5-year survival rate of 25% in Europe [2]. At present, the treatment of choice for gastric cancer is complete surgical removal of the tumour and adjacent lymph nodes. However, even after macroscopic complete removal of the primary tumour and metastatic lymph nodes, many patients with advanced disease have recurrence. The effectiveness of therapeutic approaches such as chemotherapy, hormonal therapy, and radiotherapy remains very limited. Although combination chemotherapy regimens consisting of two or three cytotoxic agents have been developed, overall survival is 10 to 13 months in patients with unresectable or metastatic gastric cancer who receive chemotherapy [3,4]. Many receptor tyrosine kinases (RTKs) have been shown to be related to tumour progression and patient outcomes in various cancers. RTK inhibitors such as human epidermal growth factor receptor (HER) have been evaluated, and some have been used to treat gastrointestinal cancers. Only trastuzumab, a monoclonal antibody against the p185HER2 protein, is now used clinically to treat unresectable or metastatic gastric cancers with HER2 overexpression. However, only 12% of patients with far advanced gastric cancer respond to trastuzumab, and the median survival time was only 16 months in patients with HER2-positive tumours who received chemotherapy with trastuzumab [5]. Other molecules associated with patient survival have therefore been investigated to identify potential targets for chemotherapy.

Erythropoietin-producing hepatocellular (Eph) receptors represent the largest known family of RTKs and are activated by interacting with cell-surface ligands, termed ephrins. Eph receptors are classified into A-type (EphA1-8 and EphA10) and B-type (EphB1-4 and EphB6) according to their interactions with ephrin ligands, which are also classified into A-type and B-type [6]. Eph receptors and ephrin ligands control cell morphology, adhesion, migration, and invasion by modifying the organization of the actin cytoskeleton and influencing the activities of integrins and intercellular adhesion molecules [7]. Combinations of Eph receptors and ephrin ligands are thought to occur in a tissue-type or cancer-type specific manner. In malignant tumours, Eph and ephrin can promote progression by activating downstream signaling pathways. The up-regulation of Eph and ephrin has been reported in various types of cancer. Altered expression patterns of Eph and ephrin correlate with tumour-promoting features such vascularization and epithelial-mesenchymal transition [8]. In gastric cancer, overexpression of EphA2, A4, and ephrinA1 has been reported by a few small studies [9,10]. We therefore examined the relation between clinical outcomes and immunohistochemical expression of EphA2, EphA4, and ephrinA1 in gastric cancer.

## Methods

### Patients

The study group comprised 222 patients with primary gastric adenocarcinomas who underwent surgery from January 2003 through December 2007 at the Department of Esophagogastric Surgery, Tokyo Medical and Dental University. This study was conducted in accordance with the Declaration of Helsinki [11] and approved by the Institutional Review Board of Tokyo Medical and Dental University. Written Informed consent was obtained from all patients in this study. Each tumour was classified according to the tumour-node-metastasis (TNM) system recommended by the International Union against Cancer. Of the 222 patients, 168 were male and 54 were female. The mean age was 64.6 years (range: 21-92 years). All patients were evaluated for recurrent disease by tumour-marker analysis or diagnostic imaging (computed tomography, ultrasonography, magnetic resonance imaging, and endoscopy) every 3 to 6 months. Patients with distant metastatic or recurrent disease received chemotherapy with S-1 alone or combined chemotherapy. Twenty patients (9%) received adjuvant chemotherapy with S-1 after radical resection. All patients were followed up until July 2012. The median follow-up was 60 months (3-111). A total of 77 (35%) patients died, 66 (30%) had recurrent disease, and 11 (5%) died of other causes.

### Immunohistochemical analysis of EphA2, EphA4, and ephrinA1 proteins

For immunohistochemical analysis, immunostaining was carried out with the use of a peroxidase-labeled polymer conjugated to secondary antibodies (Histofine Simple Stain MAX PO [MULTI], Nichirei Co., Tokyo, Japan). Polyclonal rabbit antibodies against Eph A2 (C-20, sc-924), Eph A4 (S-20, sc-921), and ephrin-A1 (V-18, sc-911) were purchased from Santa Cruz Biotechnology, Inc. (Santa Cruz, CA, U.S.A.). These antibodies have been used in other studies [9,12-14], and the specificities of EphA2 and ephrinA1 antibodies were demonstrated by immunoadsorption tests or Western blotting (excluding EphA4) [13,14]. All available hematoxylin-and-eosin-stained slides of the surgical specimens were reviewed.

For each case, representative paraffin blocks were selected for immunohistochemical studies. Three-micrometer-thick sections were cut from formalin-fixed, paraffin-embedded tissue blocks. After deparaffinization and rehydration, antigen retrieval treatment was carried out at 98°C (microwave processor, MI-77, AZUMAYA, Tokyo, Japan) for 20 min in pH 6.0, 10 mmol/L sodium citrate buffer (Mitsubishi Chemical Medience Corporation, Tokyo, Japan). Endogenous peroxidase was blocked with 3% hydrogen peroxide in methanol. Nonspecific binding was then blocked by treating the slides with 10% normal goat serum for 10 min at room temperature. The slides were incubated with primary antibodies including Eph A2 (dilution 1:100), Eph A4 (1:150), and ephrinA1 (1:200) in 1% BSA/PBS(-) overnight at 4°C. Sections were incubated with Simple Stain Max PO (MULTI) for 30 min. After three additional washes, 3,3′-diaminobenzidine tetrahydrochloride solution (Histofine Simple Stain DAB Solution, Nichirei Co., Tokyo, Japan) was applied. Sections were then counterstained with Mayer’s hematoxylin (WAKO, Tokyo, Japan). Negative controls were treated similarly, except that the antibodies were replaced by normal rabbit IgG (Santa Cruz Biotechnology, Inc.). Breast cancer tissues served as positive controls.

### Interpretation of the immunostaining results

Staining intensity was scored into four grades: 0 (none), 1 (weakly positive), 2 (moderately positive), and 3 (strongly positive). Staining extent (positive frequency) was also scored into four grades according to the percentage of stained tumour cells: 0 for complete absence of staining, 1 for <20%, 2 for 20% to <50%, and 3 for ≥50% cells. Composite scores were derived by multiplying the intensity score by the staining-extent score. For statistical analysis, composite scores of ≥4 were defined as high expression, and scores of <4 were considered low expression. Two investigators (M.K. and T.Y.), who were blinded to patients’ outcomes separately counted stained cancer cells in at least three fields per section, including the deepest site invaded by cancer cells, the surface of the lesion, and an intermediate zone. Any disagreements between the two investigators were resolved by reassessment and consensus.

### Statistical analysis

The χ^2^ test was used to test possible associations of Eph/ephrin expression with clinicopathological variables. The Mann-Whitney *U*-test was used to analyse the relation between Eph/ephrin expression and patient age. Kaplan-Meier curves were plotted to assess the effect of Eph/ephrin expression on disease-specific survival (DSS). Different DSS curves were compared using the log-rank test. Multivariate proportional Cox models were used to assess the prognostic significance of Eph/ephrin and factors associated with DSS. *P* values of less than 0.05 were considered to indicate statistical significance. Statistical analysis was done using IBM SPSS Statistics 20 software (IBM, Inc., Armonk, NY, U.S.A.).

## Results

### Immunohistochemistry of EphA2, EphA4, and ephrinA1

**Figure 1:**
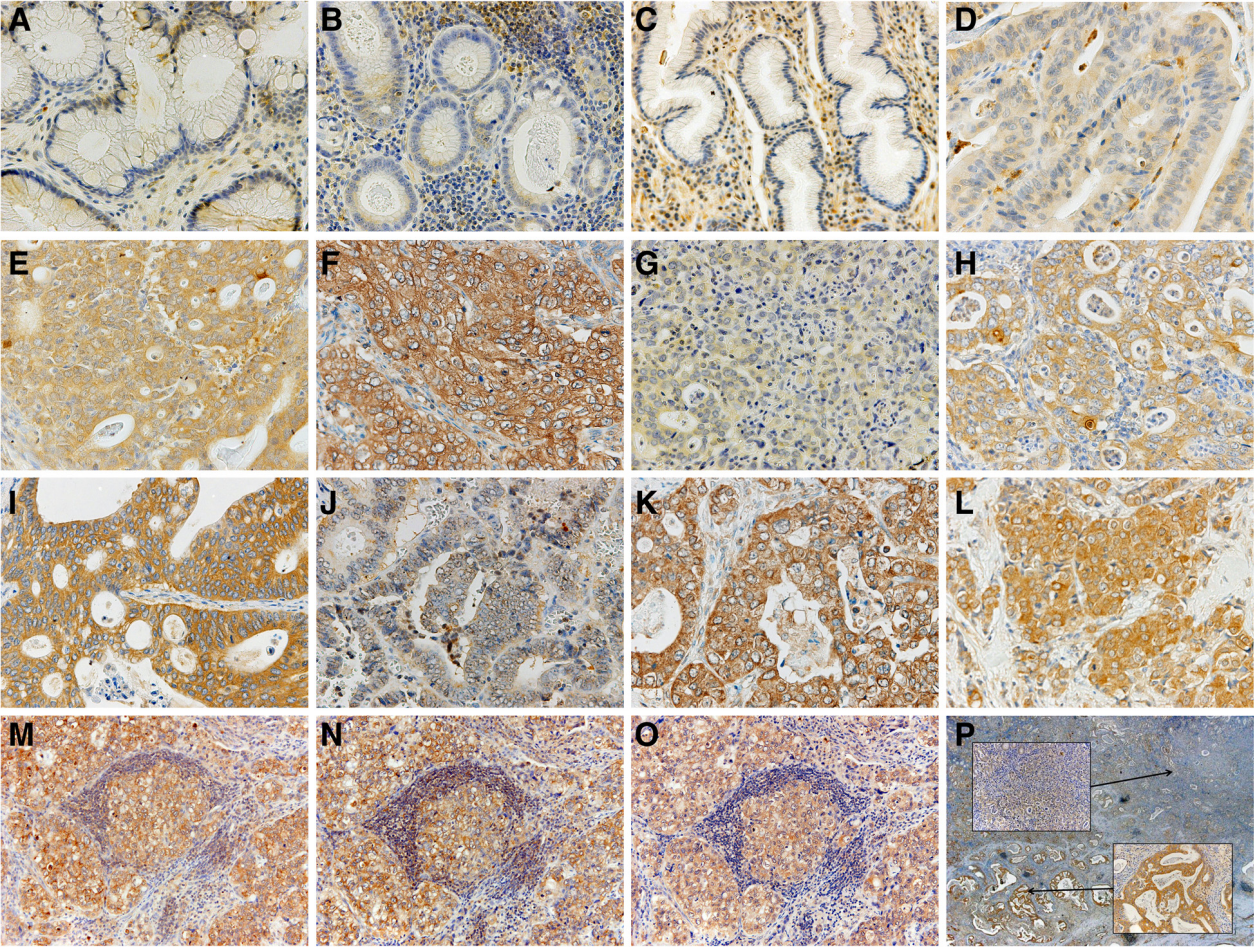
**Immunostaining for EphA2, EphA4, and ephrinA1. **Representative normal gastric epithelium and stromal cells showing no or weak immunostaining for EphA2 (**A**), EphA4 (**B**), and ephrinA1 (**C**). Representative primary gastric carcinomas showing immunostaining for EphA2 with intensity scores of 1 (**D**), 2 (**E**), and 3 (**F**), immunostaining for EphA4 with intensity scores of 1 (**G**), 2 (**H**), and 3 (**I**), and immunostaining for ephrinA1 with intensity scores of 0 (**J**) and 3 (**K**). Positive control for ephrinA1 in breast cancer tissue is shown (**L**). Representative metastatic lymph nodes showing immunostaining for EphA2 (**M**), EphA4 (**N**), and ephrinA1 (**O**). Magnification, 400× (**A**-**L**), 200× (**M**-**O**), Heterogeneous staining of EphA4 in one tumor (**P**); magnification, 20×. The upper and lower insets show EphA4 with intensity scores of 1 and 3, respectively; magnification, 200 × .

Expressions of EphA2, EphA4, and ephrinA1 were mainly observed in the cytoplasm of cancer cells (Figure 1). Expression was also noted in lymphocytes and blood endothelial cells in cancer tissue. Weak expression was found in some regions of normal epithelium. High expression of EphA2, EphA4, and ephrinA1 was found in 145 (65%), 111 (50%), and 115 (52%) patients, respectively. The numbers of patients with composite scores of 4, 5, and 6 were respectively 56 (25%), 72 (32%), and 17 (8%) for EphA2, 8 (26%), 41 (18%), and 12 (5%) for EphA4, and 79 (36%), 32 (14%), and 4 (2%) for ephrinA1. High expression of EphA2, EphA4, or ephrinA1 significantly correlated with high expression of each of the other two proteins (Table 1). On evaluation of 91 metastatic lymph nodes, high expression of EphA2, EphA4, and ephrinA1 was found in 56 (62%), 60 (66%), and 39 (43%) patients, respectively. However, only EphA4 showed a significant relation between expression in primary tumours and that in metastatic lymph nodes (p = 0.012, Table 2).

**Table 1:** Correlations among expressions of EphA2, EphA4, and ephrinA1

	**EphA4**			**ephrinA1**		
	**low**	**high**	**p**	**low**	**high**	**p**
EphA2						
low	59	18	<0.001	53	24	<0.001
high	52	93		54	91	
EphA4						
low				76	35	<0.001
high				31	80	

**Table 2:** Correlations of EphA2, EphA4, and ephrinA1 between primary tumour and metastatic lymph nodes

		**Metastatic lymph nodes**		
		**low**	**high**	**p**
	EphA2			
	low	8	12	0.87
	high	27	44	
	EphA4			
Primary	low	14	12	0.012
tumor	high	17	48	
	ephrinA1			
	low	21	15	0.85
	high	31	24	

### Relationship to clinicopathological variables

Clinicopathological variables are shown in Table 3. High expressions of EphA2, EphA4, and ephrinA1 were significantly associated with the depth of tumour invasion (T3-T4 versus T1-T2; p < 0.001, p < 0.001, and p = 0.001, respectively), lymph node metastasis (p = 0.001, p < 0.001, and p = 0.007, respectively), and tumour stage (III-IV versus I-II; p < 0.001, p < 0.001, and p = 0.001, respectively). Lymphatic and venous invasion were significantly associated with high expressions of EphA2, EphA4, and ephrinA1 (lymphatic invasion: p = 0.001, p < 0.001, and p < 0.001; venous invasion: p < 0.001, p < 0.001, and p < 0.001, respectively). Distant metastasis or recurrence was found in a significantly higher proportion of patients with high expressions of EphA2, EphA4, and ephrinA1 than in those with low expressions of these proteins (p < 0.001, p < 0.001, and p = 0.025, respectively).

**Table 3:** Correlations between the expression of EphA2, EphA4, and ephrinA1 and clinicopathological factors

		**EphA2**			**EphA4**			**ephrinA1**		
		**low**	**high**	**p**	**low**	**high**	**p**	**low**	**high**	**p**
	n	77	145		111	111		107	115	
Age										
<70	142	53	89	0.27	75	67	0.26	72	70	0.32
≥70	80	24	56		36	44		35	45	
Gender										
female	54	17	37	0.57	32	22	0.12	29	25	0.35
male	168	60	108		79	89		78	90	
Main location										
middle or lower	177	66	111	0.11	89	88	0.87	92	85	0.025
upper	45	11	34		22	23		15	30	
WHO pathological type										
differentiated	107	35	72	0.55	54	53	0.89	48	59	0.34
undifferentiated	115	42	73		57	58		59	56	
Depth of invasion										
T1/2	118	59	59	<0.001	85	33	<0.001	69	49	0.001
T3/4	104	18	86		26	78		38	66	
Lymphatic invasion										
negative	69	35	34	0.001	51	18	<0.001	47	22	<0.001
positive	153	42	111		60	93		60	93	
Venous invasion										
negative	73	39	34	0.001	55	18	<0.001	52	21	<0.001
positive	149	38	111		56	93		55	94	
Lymph node metastasis										
negative (N0)	114	54	60	0.001	77	37	<0.001	65	49	0.007
positive (N1/2/3)	108	23	85		34	74		42	66	
Stage										
I / II	142	66	76	<0.001	95	47	<0.001	80	62	0.001
III/IV	80	11	69		16	64		27	53	
Distant metastasis or recurrence										
negative	152	69	83	<0.001	99	53	<0.001	81	71	0.025
positive	70	8	62		12	58		26	44	

### Relationship to DSS

**Figure 2:**
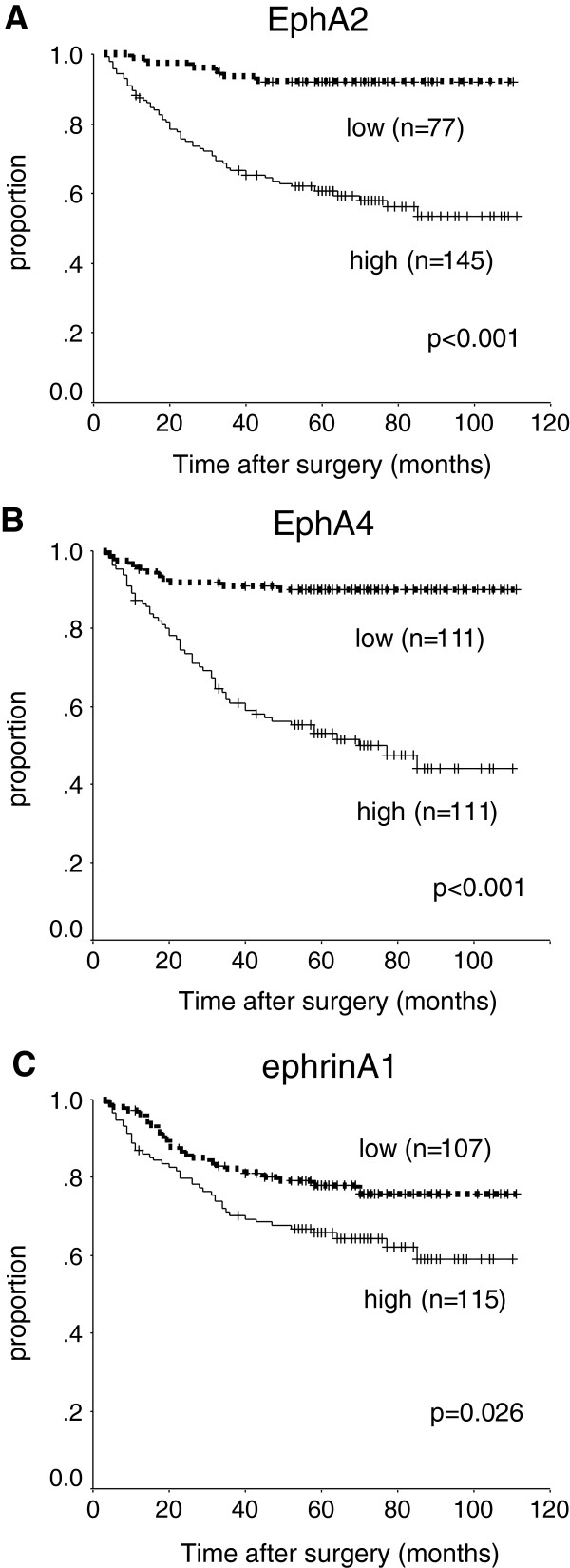
**Survival of all patients. **Kaplan-Meier curves for the disease-specific survival of patients with expression of EphA2 (**A**), EphA4 (**B**), and ephrinA1 (**C**) in the study group as a whole.

High expressions of EphA2, EphA4, and ephrinA1 were significantly associated with poorer DSS on univariate analysis (p < 0.001, p < 0.001, and p = 0.026, respectively, Figure 2). The 5-year DSS was respectively 60%, 52%, and 65% in patients with high expression of EphA2, EphA4, and ephrinA1, as compared with 92%, 90%, and 78% in patients with low expression. EphA4 was an independent predictor of DSS (HR, 2.3; 95% CI, 1.1-4.8; p = 0.028) on multivariate Cox proportional-hazards regression analysis (Table 4) adjusted for the following established clinical prognostic factors: depth of tumour (T3-T4 versus T1-T2), lymph node metastasis, and histopathological type (undifferentiated versus differentiated). EphA2 showed a trend toward being an independent prognostic factor (HR, 2.4; 95% CI, 1.0-5.8; p = 0.050).

**Table 4:** Prognostic factors in univariate and multivariate Cox proportional-hazards regression models for disease-specific survival in the study group as a whole

	**Univariate (Log-rank)**			**Multivariate**	
	**5-yr DSS(%)**	**p**	**HR**	**95%CI**	**p**
Age					
<70	72				
≥70	69	0.37			
Gender					
female	72				
male	71	0.82			
Main location					
middle or lower	74				
upper	62	0.15			
WHO pathological type					
differentiated	80		1		
undifferentiated	63	0.007	1.6	0.94-2.7	0.080
Depth of invasion					
T1/2	95		1		
T3/4	45	<0.001	4.2	1.6-11	0.003
Lymph node metastasis					
negative	95		1		
positive	47	<0.001	4.1	1.8-10	0.001
EphA2					
low	92		1		
high	60	<0.001	2.4	1.0-5.8	0.050
EphA4					
low	90		1		
high	52	<0.001	2.3	1.1-4.8	0.028
ephrinA1					
low	78		1		
high	65	0.026	0.88	0.50-1.5	0.64

### Relationship to DSS in stage II and III disease

**Figure 3:**
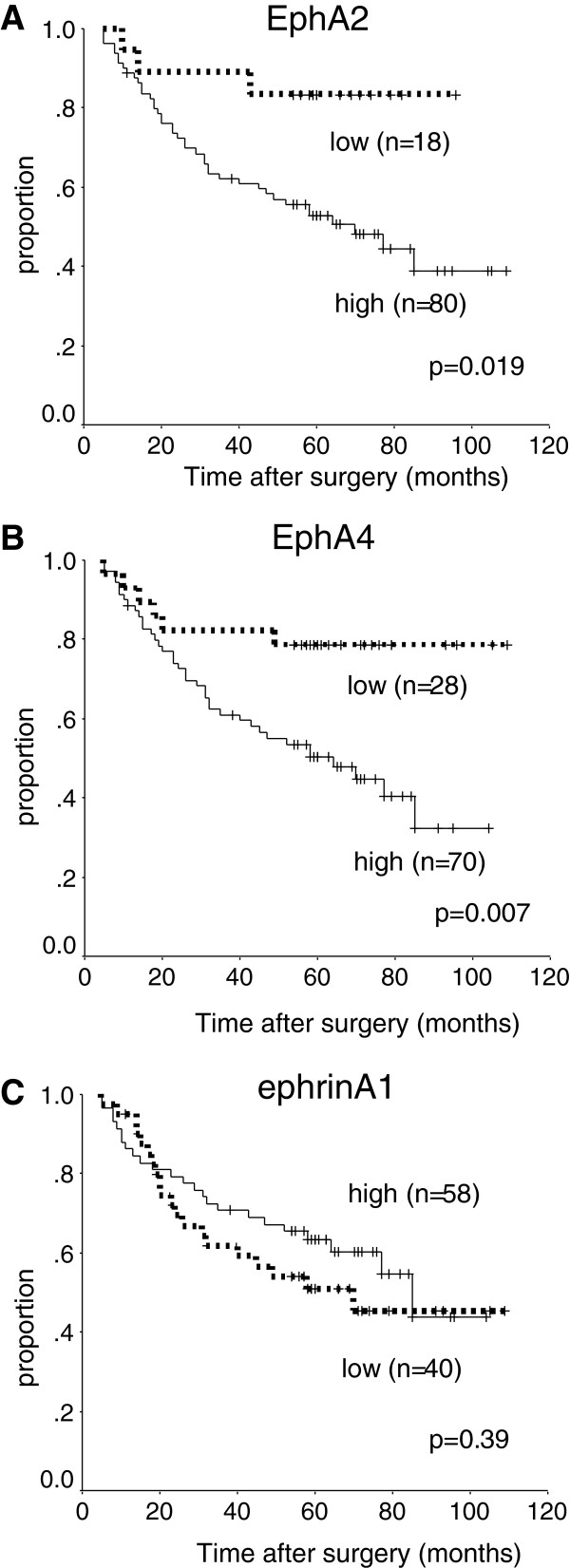
**Survival of patients in stage II and III. **Kaplan-Meier curves for the disease-specific survival of patients with expression of EphA2 (**A**), EphA4 (**B**), and ephrinA1 (**C**) who had stage II and III disease.

In patients with pathological stage II and III disease, high expression levels of EphA2 and EphA4 were significantly associated with poorer DSS on univariate analysis (p = 0.019, p = 0.007, Figure 3). In contrast, ephrinA1 was unrelated to DSS (p = 0.39). The 5-year DSS was respectively 52% and 49% in patients with high expression of EphA2 and EphA4, as compared with 83% and 79% in those with low expression. EphA4 was an independent predictor of DSS on multivariate Cox proportional-hazards regression analysis (HR, 2.6; 95% CI, 1.1-6.3; p = 0.039, Table 5) adjusted for the following established clinical prognostic factors: depth of tumour (T4 versus T1-T3), lymph node metastasis, and histopathological type. The depth of tumour invasion was classified into T4 and T1-3 tumours in this limited population because there were few T1 and T2 tumors, and the difference in DSS was more statistically significant between T4 and T1-3 than that between T3-4 and T1-2 on univariate analysis. EphA2 was not a significant prognostic factor in stage II and III gastric cancer.

**Table 5:** Prognostic factors in univariate and multivariate Cox proportional-hazards regression models for disease-specific survival in patients with stage II and III disease

		**Univariate (Log-rank)**			**Multivariate**	
	**n**	**5-yr DSS(%)**	**p**	**HR**	**95%CI**	p
Age						
<70	65	54				
≥70	33	64	0.63			
Gender						
female	22	64				
male	76	56	0.49			
Main location						
middle or lower	78	61				
upper	20	44	0.24			
WHO pathological type						
differentiated	39	69		1		
undifferentiated	59	50	0.077	1.7	0.86-3.3	0.13
Depth of invasion						
T1-3	48	85		1		
T4	50	32	<0.001	5.5	2.5-12	<0.001
Lymph node metastasis						
negative	20	89		1		
positive	78	49	0.004	3.0	0.92-9.9	0.070
EphA2						
low	18	83		1		
high	80	52	0.019	1.7	0.52-5.7	0.38
EphA4						
low	28	79		1		
high	70	49	0.007	2.6	1.1-6.3	0.039
ephrinA1						
low	40	51				
high	58	62	0.39			

## Discussion

Our results suggest that high expression of EphA4 and EphA2 may play critical roles in tumor progression, metastasis, and outcomes in gastric cancer. EphA4 was an independent prognostic factor in gastric cancer, even in advanced disease requiring adjuvant chemotherapy after resection to prevent recurrence. Our findings are consistent with those of a previous study showing that overexpression of EphA4 on immunohistochemical analysis is an independent predictor of overall survival in gastric cancer [9]. Overexpression of the EphA4 gene in colorectal primary tumors has been found to be associated with liver metastasis, although expression levels of this gene did not correlate with any other clinicopathological factor and did not differ between cancer tissue and adjacent normal mucosa [10]. In breast cancer, elevated RNA expression of EphA4 had significant prognostic value, as did EphA2, EphA7, and EphB4 [15]. High gene expression of EphA4 has been linked to overexpression of the protein in gastric cancer [9], whereas EphA4 gene amplification has not. Some activated signaling pathways seem to be involved in tumour progression. Activated EphA4 and EphA2 have been shown to trigger the activation of RhoA, which ultimately led to reinitiation of migration in a different direction in a prostate cancer cell line [16]. EphA4 forms a heteroreceptor complex with fibroblast growth factor receptor 1 (FGFR1) in glioma cells, and the EphA4-FGFR1 complex potentiates FGFR-mediated downstream signaling [17]. EphA4-ephrinA3 pathway has been considered a promising target in pancreatic cancer cells [12]. EphA4 can interact with Ephrin-A as well as Ephrin-B [18,19], although EphA receptors bind mainly to ephrin-A ligands (A1-A5), and EphB receptors bind to transmembrane ephrin-B ligands (B1-B3). In the present study, other ligands for EphA4 (except for ephrinA1) were not investigated. On the other hand, even tumor-suppressive activation of EphA4 was evident in another study. EphA4 has been reported to inhibit downstream Rac, which promotes cell migration, through chimaerin binding to EphA4 in response to ephrinA1 stimulation [20].

EphA2 is thought to down-regulate cell growth and migration in normal epithelium [21]. Interestingly, EphA2 is highly expressed in a variety of cancers, including breast [22], lung [23], prostate [24], urinary bladder [25], ovarian [26], esophageal [27], pancreatic [28], and colorectal cancer [29]. Overexpression of EphA2 is associated with tumour progression or poor patient survival. The expression of ephrinA1, known to be a major ligand of EphA2, has been studied in various cancers. Expressions of both EphA2 and ephrinA1 on the basis of the mRNA level or immunohistochemical analysis have been demonstrated to be higher in gastric cancer than in normal tissue, and EphA2 was significantly associated with poor survival, whereas ephrinA1 was not [13]. EphrinA1 expression has been significantly associated with EphA2 expression, although ephrinA1 was not an independent prognostic factor in several types of cancer [27,30,31]. Our findings are in accord with the results of these previous studies. In gastric cancer cell lines that express EphA2, stimulation of EphrinA1 decreases EphA2 protein expression, but increases EphA2 phosphorylation [14]. Also in non-tumour cells, EphA2 was degraded by Cbl binding to ephrinA1 [32]. On the other hand, expression of EphA2 and ephrinA1 has been confirmed in both the vasculature and tumor cells in cancer tissues, and EphA2 might be required for angiogenesis in an *in vitro* model [33]. The interaction of Eph receptor and ephrin ligand expressed on vasculature cells might be associated with angiogenesis in tumours. In the present study, EphA2, ephrinA1, and even EphA4 were also expressed in vascular endothelial cells existing in cancer tissue.

Gene expression or quantitative assessment is important and necessary to confirm the outcomes of this study, although mRNA expression levels of EphA2, EphA4, and ephrinA1 were shown to be higher in gastric cancer tissue than in non-cancerous gastric tissue by other investigators [9,13]. Many pathological and molecular assays suggest that gastric cancer is a heterogeneous disease. Analysis of one small part of a specimen by techniques such as tissue microarray may not convey the entire picture of heterogeneous diseases such as gastric cancer, consisting of many scattered tumour cells. Staining for EphA2, EphA4, and ephrinA1 often differed between the lesion surface and sites of deep invasive or between differentiated and undifferentiated portions of the same sample in the present study. We also evaluated the expressions of EphA2, EphA4, and ephrinA1 in metastatic lymph nodes. To our knowledge, Eph and ephrin in metastatic sites of gastric cancer have not been investigated previously. Expressions of EphA2 and ephrinA1 differed between primary tumor and lymph node metastasis and correlated significantly with the expression of only EphA4. This discrepancy may have been caused by methodological issues, although gene analysis by extraction from metastatic lymph nodes may be difficult owing to the presence of scattered tumour cells in innumerable lymphocytes. Differences in EphA2 or ephrinA1 expression among tumour sites can make chemotherapy against these molecular targets challenging.

Various molecules targeting Eph and ephrin have been developed. A small molecule that inhibits binding of ephrin to EphA2 and EphA4 has been identified [19], and such inhibitors might be effective against advanced or metastatic gastric cancer. Small interfering RNA-mediated inhibition of EphA2 has been reported to retard tumour growth and inhibit metastasis in an *in vivo* study of pancreatic adenocarcinoma [34]. However, Eph and ephrin are known to have bidirectional signaling in cancer cells. Therefore, EphA2 agonists most likely enhance tumor suppressor signaling pathways and receptor degradation in cancer cells, but promote tumour angiogenesis [8].

## Conclusion

EphA4 plays an important role in tumour progression and clinical outcomes, similar to EphA2, in patients with gastric cancer. EphA4 is an independent prognostic factor in stage II and III gastric cancer, stages that usually require adjuvant chemotherapy. The expression of EphA4 in primary tumours significantly correlated with that in metastatic lymph nodes. EphA4 may be a promising target for monoclonal antibody therapy in patients with gastric cancer.

## Competing interests

We declare that we have no competing interests.

## Authors’ contributions

KM, MI, and KS were responsible for drafting the manuscript. KM, KK, and YT contributed to immunohistochemistry. MI and KK contributed to analysis and interpretation of data. All authors read and approved the final manuscript.

## Pre-publication history

The pre-publication history for this paper can be accessed here:

http://www.biomedcentral.com/1472-6890/13/19/prepub
